# The Effect of Narrative on Physical Activity via Immersion During Active Video Game Play in Children: Mediation Analysis

**DOI:** 10.2196/17994

**Published:** 2020-03-31

**Authors:** Caio Victor Sousa, Austin Fernandez, Jungyun Hwang, Amy Shirong Lu

**Affiliations:** 1 College of Arts, Media, and Design, Bouvé College of Health Sciences Health Technology Lab Northeastern University Boston, MA United States; 2 Stanford University Medical Center Department of Medicine Palo Alto, CA United States

**Keywords:** video games, exercise, fitness trackers, narration, physical activity, exercise

## Abstract

**Background:**

Active video games (AVGs) can increase physical activity (PA) and help produce higher physiological expenditure. Animated narrative videos (NVs) possess unique immersive and motivational properties. When added to AVGs, they have been found to increase moderate-to-vigorous physical activity (MVPA) as opposed to the original no video condition. However, there is no evidence of whether that was due to the NV or the addition of an animated video to an AVG.

**Objective:**

This study aimed to investigate the differential effect of adding an NV versus a nonnarrative video (NNV) to an AVG on PA parameters and physiological responses and to explore the mediating role of immersion.

**Methods:**

A total of 22 children aged 8 to 12 years were randomly assigned to the NV or NNV condition. They were instructed to play an AVG (on Xbox Kinect) for as long as they wanted. We used accelerometers to estimate the time spent (in minutes) in MVPA. Heart rate (HR) and rate of perceived exertion (RPE) were measured before, during, and after the AVG play session. The participants then reported their experience of narrative immersion via a questionnaire.

**Results:**

The NV group had significantly higher narrative immersion (mean 3.50, SD 0.55 vs mean 2.91, SD 0.59; *P*=.03) and MVPA (mean 19.46, SD 13.31 vs mean 7.85, SD 5.83; *P*=.02) than the NNV group. Narrative immersion was positively correlated with MVPA (*r*=0.52; *P*=.01) and average HR during AVG (*r*=0.43; *P*=.05). Mediation analysis indicated that narrative immersion mediated the effect of NV (NV vs NNV) on MVPA (direct effect: beta=7.51; *P*=.01). The indirect effect was that NV was positively correlated with the mediator variable narrative immersion (beta=.59; *P*=.03), which was itself marginally associated with MVPA (beta=6.95; *P*=.09); when narrative immersion was included in the model, the regression coefficient was attenuated.

**Conclusions:**

AVG with added narratives elicits more narrative immersion, resulting in more minutes in MVPA. Narrative immersion served as a mediator between NV and MVPA via its elicitation of an elevated HR without increasing RPE. The inclusion of immersive narratives in AVG could be helpful for inducing MVPA, to enhance AVG engagement without additional exertion.

## Introduction

In the United States, children spend an excessive amount of time with screen media [1]. Their daily time spent in sedentary video games has tripled over the past decade [2]. Physical activity (PA) in children is identified as one of the main methods for reducing the risk of obesity [3], cardiovascular disease [4], and type 2 diabetes [5]. Obese children aged less than 12 years are highly likely to become obese young adults [6].

A narrative is defined as any two or more events arranged in a temporal order [7] with unique, immersive, and motivational properties to encourage continuous active video game (AVG) play [8]. The absorption in a storyline integrates attentional allocation with imagery and feelings related to the narrative as opposed to nonnarrative. A loss of self-awareness is combined with mental construction of the narrative reality [9]. This attentional allocation is measured by narrative immersion [10]. Immersion is a term adapted from media research, defined as “a state in which the reader or player becomes absorbed in the narrative world” [10,11], that is, when one’s perception is directed toward a mediated world and away from the physical world [9,12]. On the other hand, nonnarratives consist of *arguments, reasoning, claims, and so forth* [13] that are overtly persuasive messages that do not create alternative worlds for individuals to enter, making them less likely to engage in mental imagery and emotion [9].

Narratives have been postulated to promote continuous gameplay [14]. AVGs emerge as an additional opportunity to increase children’s PA levels and reduce sedentary media usage [15,16] because AVGs are able to elicit moderate-to-vigorous physical activity (MVPA) [17,18]. AVGs feature player movement similar to *real-life* exercise participation [19,20]. Previous research demonstrated that children playing an AVG sports game session have similar energy expenditure as other traditional activities such as dancing or cycling [21]. However, children do not play AVG for sufficiently long durations to have an impact on their PA levels [20]. Thus, the development of innovative methods to increase children’s adherence and engagement to AVG is warranted. Although narratives appear in some health games [22], few AVGs capable of achieving MVPA have incorporated them [8].

The effect of narrative videos (NV) versus nonnarrative videos (NNV) has been studied extensively in health communication, behavioral intervention, and science communication [10,23], but no prior research has induced MVPA in AVG. A recent study [24] compared the effect of adding a narrative animated video to an AVG play session with the same session without video and reported that the children who watched the narrative took significantly more steps during the AVG play. Similarly, this effect was replicated recently [25]. In this case, the addition of a narrative to an AVG increased the MVPA by more than 50% compared with the same game condition without the narrative. Nevertheless, it is possible that the positive effect was merely because of the addition of an animated video, not the narrative itself. Although only NVs should have the immersive properties to increase engagement and motivation, there is no evidence that an NNV (documentary-like without stories) could also increase immersion, and there is no evidence of how MVPA increases during AVG.

Thus, this study aimed to compare the differential effect of adding an NV versus an NNV to an AVG session on PA parameters and the physiological responses to these two situations and to explore the mediation effect of immersion between *narrative versus nonnarrative* and MVPA in children aged 8 to 12 years.

## Methods

### Ethics Protocol

The Northeastern University’s institutional review board (IRB) approved the research protocol. Parents provided written informed consent, and children provided written informed assent. Consent and assent forms were both delivered to families of eligible children via email, and the paper forms were collected when parents brought the children to a university laboratory for the study. Data were collected between July 2018 and October 2018. Two research assistants (RAs) conducted the data collection with all participants.

### Study Population

Children were recruited from a large, diverse, urban neighborhood in the northeast United States. RA placed printed flyers in the neighborhood afterschool program rooms and local public libraries. Participants from a previous research pool who were interested in this study were also contacted.

Inclusion criteria required the participants to (1) be aged between 8 and 12 years, (2) speak and understand English, (3) have not previously played the selected AVG, and (4) have no physical limitations with respect to AVG play. Exclusion criterion was the inability to complete an AVG session because of a medical condition or physical limitation.

The 8- to 12-year age group was targeted because children younger than 8 years have cognitive limitations in responding to survey questions [26] and children older than 12 years have entered early adolescence and will be subject to many physical, mental, emotional, and social changes that may make their needs and responses different from those of younger children [27]. In addition, without intervention, obese children in this age group (8-12 years old) are highly likely to become obese young adults [6].

### Media Materials and Procedures

#### Experimental Videos

Both NV and NNV had a duration of approximately 11 min and presented the same kind of information on the benefit of PAs. The NNV was a narrated scientific documentary–like video about the benefits of PA featuring cartoon characters, including alternated male and female narrators. The NV tells a science fiction story about 2 main characters (boy/girl and player) with special powers enhanced by exercise and PA. The character/player needs to enhance their power to survive a postapocalyptic world. The NV plot has been developed with children-friendly design guidelines for MVPA motivation [28,29] and has been found to be liked by children in this age group [30].

#### Active Video Game

We used the *Kung Fu for Kinect* AVG (Virtual Air Guitar Company), which involves whole-body movements via a Kinect sensor on an Xbox One console (Microsoft Inc). A preliminary examination of the AVG also indicated that both the NV and the NNV would be seamlessly matched with the game. While playing the AVG, the participant could see his/her own body on the screen and fought enemies using his/her own moves in a 2-dimensional fighting adventure environment. When different enemies appeared on the screen, the participant engaged them with a variety of intermittent and spontaneous movement patterns and skills, such as jumping, punching, and kicking [17]. We set the difficulty level as *easy* because we did not want the first time a child played the AVG to be difficult, and we found the level to be exercising enough to make the participants move. We set up a playing time limit of 45 min to avoid exhaustion, but the participants could stop at any point after they played at least one level (the first level could last between 1 and 5 min). We determined that a level ended whether a player won or did not win. Participant players could choose to continue trying that level if they had lost or progress to the next level if they had won.

### Demographic Information and Anthropometrics

After signing an informed consent form, parents completed a questionnaire about their demographic and socioeconomic information (gender, race, household income, education, and number of adults and children in the household).

Children’s height (nearest 0.1 cm) and weight (nearest 0.1 kg) were measured using a stadiometer (ShorrBoard, Weight and Measure LLC) and a calibrated scale (SECA GmbH), which were then used to calculate BMI. Children’s age- and sex-specific BMI percentiles were obtained from the Centers for Disease Control and Prevention BMI-for-age growth charts [27].

A random list function (MS Office Excel 2019) was used to randomize eligible children to watch either an NV or an NNV before AVG play.

### Accelerometer and Physiological Measures

The participants had a full and detailed explanation of the study procedures. A Polar heart rate (HR) monitor (Polar) was attached below the participant’s chest, and an ActiGraph GT3x (ActiGraph) was attached to their nondominant hip.

With all apparatus set, participants first sat comfortably before a television screen to watch an 11-min animated video (either NV or NNV). The RA stayed behind a curtain and remotely turned on the AVG console after the video. Children were asked to play for as long as they wanted.

Rate of perceived exertion (RPE) was assessed with Borg scale before and after the AVG play session. HR and PA were monitored during the entire AVG play session using the attached devices. The ActiGraph accelerometer device has been widely used for assessing individual levels of PA across different age groups, including children and young adults [17,31,32]. The ActiGraph triaxial accelerometers measure accelerations from the subject’s intensity and frequency of movement in 3 individual axes (anterior-posterior, vertical, and medial-lateral) and were initialized at 30 Hz sampling. The ActiLife software version 6.13.2 (ActiGraph) was used to download the data from the activity monitors and to convert acceleration data into the 3 axes activity counts, which quantify the amplitude and frequency of detected accelerations at a 1-second epoch dataset. A 1-second epoch has been found to be the most appropriate epoch length to detect short bursts of intense PA and may be the best choice for data processing and analysis in AVG studies examining intermittent PA [31]. We used Evenson et al’s [33] activity cut points to estimate the amount of time spent in light (26-573), moderate (574-1002), and vigorous (≥1003) PA.

### After-Play Questionnaire

After the AVG session, participants completed questionnaires to assess narrative immersion and social desirability. Narrative immersion was assessed using a 9-item Likert-type scale (response range 1-5) [11], where 1=disagree and 5=agree. Sample items included “I could see myself in this story,” “I wanted to see how the story ended,” and “The story influenced my feelings.”

Social desirability (the tendency of the reporting person to give positive self-descriptions) was assessed using a 9-item Likert-type scale (response range 1-5) [34], where 1=disagree and 5=agree. Sample items included “I like everyone I know,” “I tell the truth every single time,” and “I never say thing I should not.” Social desirability of response has been observed in the self-reports of both adults and children. As this may impact the accuracy of the evaluative responses of AVGs, the validity of the study may be affected. The instrument has shown good reliability and validity in children across a variety of ethnic groups.

After completing the questionnaires, each participant received a US $25.00 gift card and was thanked for their participation.

### Statistical Analysis

Normality and homogeneity of the data were checked using Kolmogorov-Smirnov and Levene tests, respectively. Independent *t* tests were performed to detect between-group differences in age and anthropometrics. Repeated measures analysis of variance was used to detect within- and between-group interactions for HR and RPE. The reliability of the questionnaires was assessed by using Cronbach alpha. In addition, Pearson correlation coefficients were calculated to detect the associations between the variables.

The mediation effects were tested for any associations we detected between the animated video condition and MVPA. The SPSS macro PROCESS was used for this analysis [35]. This macro uses bootstrapping to estimate the mediated effect and bias-corrected accelerated 95% CI of the effect. For each simulated sample, the macro estimates the product of paths *b* (from the independent variable to the mediator) and *c* (from the mediator to the dependent variable). These products represent a distribution approximating the sampling distribution of the indirect effect in the population. This technique directly tests the significance of the indirect effect (the *b×c* product). This technique is considered superior to the causal steps method, which does not directly test a mediated effect [36]. This model determines whether the association between the video type (independent variable) and MVPA (dependent variable) was mediated by narrative immersion (mediation variable).

A priori sample size was calculated to detect a significant difference between the 2 conditions, with 95% power (1−beta) to detect a small effect size (EF=0.3). Owing to some sample loss, the final sample size (n=22) provided a power of 92% (1−beta) for a large EF (ie, 0.8). Although less than expected, it was still above 80%. We set the significance level at 5% (alpha≤.05). We performed our analyses using Statistical Software for the Social Sciences (IBM SPSS version 25).

## Results

### Demographics and Anthropometrics

A total of 22 participants (NV: 12 vs NNV: 10) completed the required research protocol, and none of the participants dropped out. Approximately half of the participants were minority children, and their parents were college graduates (Table 1). We tested our randomization and found that children from NV and NNV groups were not statistically different in age, height, weight, BMI percentile, or socioeconomic status (*P*s>.11).

**Table 1:** Age, anthropometrics, and demographic characteristics of the sample.

Variables		Narrative (n=12)	Nonnarrative (n=10)	*P* value
Age (years), mean (SD)		9.58 (1.16)	10.30 (1.34)	.19
Height (cm), mean (SD)		141. 3 (9.99)	146.5 (7.98)	.19
Weight (kg), mean (SD)		33.80 (7.84)	40.27 (9.92)	.11
BMI percentile, mean (SD)		45.08 (25.97)	56.80 (35.14)	.40
**Sex, n (%)**				
	Male	8 (67)	7 (70)	N/A^a^
	Female	4 (33)	3 (30)	N/A
**Race, n (%)**				
	White	7 (64)	4 (40)	N/A
	Black or African American	1 (9)	2 (20)	N/A
	Asian	2 (18)	2 (20)	N/A
	Other	1 (9)	0 (0)	N/A
	Prefer not to answer	0 (0)	2 (20)	N/A
**Highest education in the household, n (%)**				
	High school graduate or General Educational Development	2 (17)	0 (0)	N/A
	Some college	1 (8)	1 (10)	N/A
	College graduate	5 (42)	6 (60)	N/A
	Postgraduate	4 (33)	3 (30)	N/A
**Household income (US $), n (%)**				
	$20,000-$39,000	3 (27)	2 (22)	N/A
	$60,000-$79,000	1 (9)	2 (22)	N/A
	$80,000-$100,000	2 (18)	0 (0)	N/A
	>$100,000	5 (46)	5 (56)	N/A
	Prefer not to answer	1 (9)	1 (11)	N/A
Number of adults in the household, mean (SD)		2.17 (0.71)	2.00 (0.47)	.52
Number of children in the household, mean (SD)		2.67 (0.45)	2.70 (1.06)	.95

^a^N/A: not applicable.

### Immersion and Social Desirability

**Figure 1:**
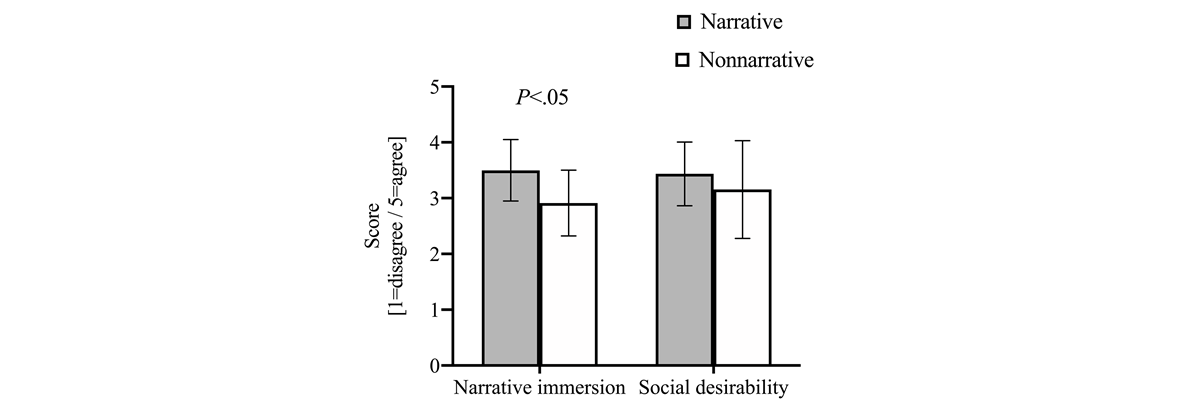
Comparison of narrative immersion between narrative videos and non-narrative videos adjusted by social desirability.

All constructs were averaged to form their respective indices after Cronbach alphas were calculated. Narrative immersion had a Cronbach alpha of .62. We found a statistically significant difference in immersion between NV and NNV groups (mean 3.5, SD 0.6 vs mean 2.9, SD 0.6; *P*=.04); we did not identify any differences for social desirability (mean 3.4, SD 0.6 vs mean 3.2, SD 0.9; *P*=.40). Our estimate of Cronbach alpha for the social desirability questionnaire for our participants was .84 (Figure 1).

### Physical Activity and Xbox Data

The PA data from the accelerometers indicated a difference in MVPA between the NV and NNV groups, although we did not identify any differences for the total playing time or the number of levels played between the NV and NNV groups (Table 2).

**Table 2:** Physical activity and active video game data.

Variables		Narrative (n=12), mean (SD)	Nonnarrative (n=10), mean (SD)	*t* test (*df*)	*P* value
**Physical activity**					
	Vector magnitude (counts)	86.2 (33.8)	64.2 (13.7)	2.060 (20)	.06
	Moderate-to-vigorous physical activity (minutes)	19.5 (13.3)	7.9 (5.8)	2.725 (20)	.02
	Total steps	1461 (838)	1049 (549)	1.383 (20)	.18
**Xbox playing data**					
	Total play time (minutes)	33.3 (14.1)	34.3 (11.5)	0.174 (20)	.86
	Number of levels	9.8 (3.7)	10.2 (2.6)	0.304 (20)	.76

### Heart Rate and Rate of Perceived Exertion Responses

**Figure 2:**
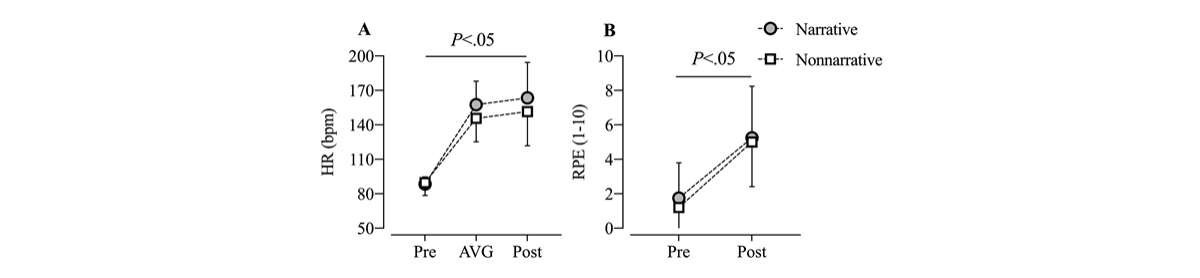
Heart rate and rate of perceived exertion during (only heart rate) and after active video game. AVG: active video game; HR: heart rate; RPE: rate of perceived exertion.

Participants’ HR and RPE responses to AVG showed a significant time effect for both NV and NNV (*Ps*<.01), although we did not identify either a group effect or a time × group effect for HR (group: *P*=.30; time × group: *P*=.17) or RPE (group: *P*=.64; time × group: *P*=.79; see Figure 2).

### Correlation Analysis

Narrative immersion was positively and moderately correlated with MVPA (*r*=0.52; *P*=.01) and average HR during AVG (*r*=.43; *P*=.05). MVPA was also positively and moderately correlated with total time playing AVG (*r*=0.56; *P*=.01) and average HR during AVG (*r*=0.60; *P*=.001; see Table 3).

**Table 3:** Correlation matrix of narrative/game scales, physical activity, and heart rate.

Variables	Immersion		Playing time		Number of levels		MVPA^a^		VM^b^	
	*r*	*P* value	*r*	*P* value	*r*	*P* value	*r*	*P* value	*r*	*P* value
Playing time	0.02	.92	N/A^c^	N/A	N/A	N/A	N/A	N/A	N/A	N/A
Number of levels	0.08	.73	*0.91* ^d^	*.001*	N/A	N/A	N/A	N/A	N/A	N/A
MVPA	*0.52*	*.01*	*0.56*	*.01*	0.53	.12	N/A	N/A	N/A	N/A
VM	*0.59*	*.001*	0.42	.06	*0.44*	*.04*	*0.81*	*<.001*	N/A	N/A
Heart rate (active video game)	*0.43*	*.05*	0.29	.19	0.18	.42	*0.60*	*<.001*	*0.74*	*<.001*

^a^MVPA: moderate-to-vigorous physical activity.

^b^VM: vector magnitude.

^c^N/A: not applicable.

^d^Italicized values denote significant correlation.

### Mediation Analysis

**Figure 3:**
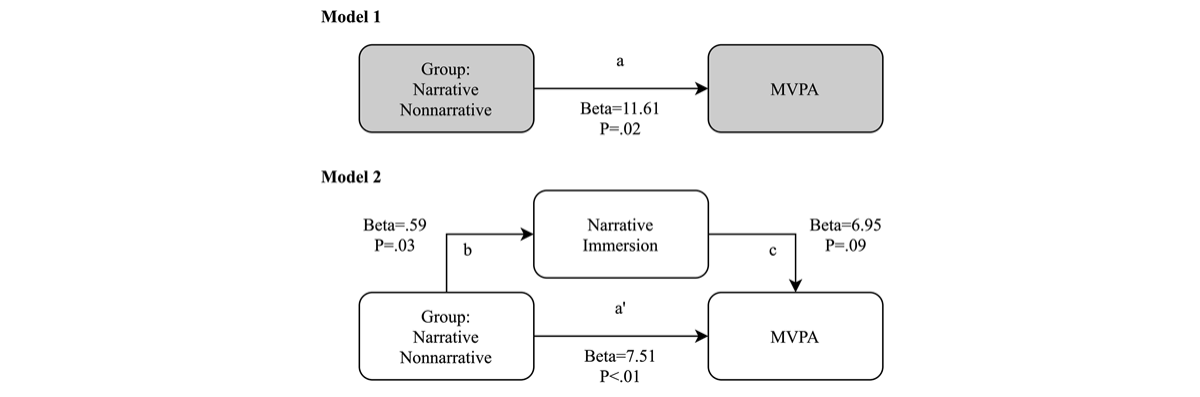
Mediation model of the relationship between narrative immersion and minutes in moderate-to-vigorous physical activity during active video game play. MVPA: moderate-to-vigorous physical activity.

The mediation analysis to test whether narrative immersion acted as a mediator variable between animated NV (independent variable) and MVPA (dependent variable) is shown in in Figure 3. Our results indicated that the effect of animated NV on MVPA was mediated by narrative immersion. In the first step (model 1: nonmediated direct effect), the regression coefficient of animated NV on MVPA indicated a positive association (beta=11.61; *P*=.02; see step *a* in Figure 3). In the second regression step (model 2: mediated direct and indirect effect), animated NV was positively related (beta=.59; *P*=.03) to narrative immersion (step *b*). In the last regression model, narrative immersion was marginally positively associated with the dependent variable (beta=6.95; *P*=.09); when narrative immersion was included in the model, the regression coefficient was significant (indirect effect: steps *b* and *c*), but the relationship was slightly attenuated (step *a’*: beta=7.51; *P*=.01). Finally, the indirect effect (step a’ plus b and c) was significant, confirming the mediation role of MVPA in this model (Figure 3).

## Discussion

### Principal Findings

This is the first study to compare NV and NNV added to an AVG play session. The main finding of this study is that AVGs with added narratives elicited more narrative immersion and narrative engagement, leading to more minutes in MVPA. More importantly, narrative immersion acted as a mediator between narratives and MVPA for AVG, also increasing HR after play without more RPE. Nevertheless, it is noteworthy that there is no interaction effect (group×time) for HR; both the groups increased their HR across time, but there are no differences between the groups. This suggests that although the addition of narratives did not result in longer periods of playtime, the players played the narrative versions of AVGs with more bodily engagement and movement, which increases their physiological response but does not necessarily increase their perceived exertion in the task. As a result, the inclusion of good immersive narratives could be a key factor in inducing children’s MVPA play time, enhancing their engagement in AVG without incurring negative physiological feelings such as exertion.

This is the first report showing a mediation effect of narrative immersion on MVPA. The mediation results showed that immersion is a key factor in increasing MVPA during AVG play. Considering that story immersion is an integrative process in which the cognitive and affective resources of the player are concentrated in the task [14], it is reasonable to hypothesize that the NV elicited more game engagement, more commitment to the game tasks, and more movements, thus increasing their PA and physiological demand, without necessarily increasing their exertion. Other AVGs (without narratives) have been shown to not sufficiently motivate children [37], which may be because of increased exertion and boredom. With narrative animations, it may be possible to prevent children from choosing sedentary games and instead motivate them to play AVGs, thus increasing PA.

Previous evidence indicated that narratives’ immersive properties could make children less self-aware of their physical effort during an AVG when they are immersed into the narrative [9], the very concept of immersion [10]. Thus, they can focus more on the outcome of the AVG play instead of the potential uncomfortable feelings associated with the exercise. Therefore, narrative immersion could be an effective method to promote healthier MVPA behaviors in young children [10] and older children [23].

Narratives enabled children to increase immersion and thus MVPA when playing AVGs. The finding that HR (during AVG), but not RPE, is moderately correlated with narrative immersion and MVPA suggests that despite the cardiovascular demand, being more physically active while being immersed in a narrative during an AVG does not increase the perceived exertion. If players perceived more exertion after AVG play, they might be discouraged from playing it again or, worse, engage in sedentary gameplay instead of active gameplay. In other words, children did not feel more tired after playing an AVG with narratives and spent more time participating in MVPA during the play instead.

### Limitations

The results are not without limitations. The small size of our study population (n=22) only allowed us to detect a large EF (*d*>0.8), and small and moderate differences may have been undetected. Most (60%-70%) of our participants were male. Children’s fitness level before the AVG play was not assessed. The laboratory setting for AVG playing may limit the external validation of the results.

### Conclusions

Narrative immersion acts as a key mediator in increasing a player’s MVPA in AVG after watching a narrative animated video. An NV also induced more MVPA with an increased HR after playing but not RPE. Future research should be conducted to investigate the chronic effect of narratives within AVGs on physiological parameters, PA, and playing time in children.

## Abbreviations

AVG: active video game
EF: effect size
HR: heart rate
IRB: institutional review board
MVPA: moderate-to-vigorous physical activity
NNV: nonnarrative video
NV: narrative video
PA: physical activity
RA: research assistant
RPE: rate of perceived exertion
