# Correction: The Effect of Narrative on Physical Activity via Immersion During Active Video Game Play in Children: Mediation Analysis

**DOI:** 10.2196/20134

**Published:** 2020-05-28

**Authors:** Caio Victor Sousa, Austin Fernandez, Jungyun Hwang, Amy Shirong Lu

**Affiliations:** 1 College of Arts, Media, and Design, Bouvé College of Health Sciences, Health Technology Lab, Northeastern University Boston, MA United States; 2 Stanford University Medical Center, Department of Medicine Palo Alto, CA United States

In the original published paper “The Effect of Narrative on Physical Activity via Immersion During Active Video Game Play in Children: Mediation Analysis” (J Med Internet Res 2020;22(3):e17994), the authors noticed an error in the Abstract and Table 2. This was caused by an updated analysis not being accurately reflected in the manuscript. The Abstract and Table 2 listed incorrect values of moderate-to-vigorous physical activity (MVPA) for the narrative (NV) group.

The value in the original Abstract section was initially listed as:

The NV group had significantly higher narrative immersion (mean 3.50, SD 0.55 vs mean 2.91, SD 0.59; *P*=.03) and MVPA than the NNV group (mean 20.11, SD 13.75 vs mean 7.85, SD 5.83; *P*=.02).

The correct value is:

The NV group had significantly higher narrative immersion (mean 3.50, SD 0.55 vs mean 2.91, SD 0.59; *P*=.03) and MVPA (mean 19.46, SD 13.31 vs mean 7.85, SD 5.83; *P*=.02) than the NNV group.

Furthermore, in Table 2, the “Narrative (n=12), mean (SD)” value for “Moderate-to-vigorous physical activity (minutes)” was originally listed as:

19.4 (13.3)

The correct “Narrative (n=12), mean (SD)” value for “Moderate-to-vigorous physical activity (minutes)” in Table 2 is:

19.5 (13.3)

The changes were not significant, and do not affect the overall findings of the paper.

The authors also want to acknowledge FableVision Studios contribution. The following sentence has been added to the Acknowledgments:

FableVision Studios produced the narrative video for this project.

The correction will appear in the online version of the paper on the JMIR website on May 28, 2020, together with the publication of this correction notice. Because this was made after submission to PubMed, PubMed Central, and other full-text repositories, the corrected article has also been resubmitted to those repositories.

